# A review of the new HGNC gene family resource

**DOI:** 10.1186/s40246-016-0062-6

**Published:** 2016-02-03

**Authors:** Kristian A Gray, Ruth L Seal, Susan Tweedie, Mathew W Wright, Elspeth A Bruford

**Affiliations:** European Bioinformatics Institute (EMBL-EBI), Wellcome Genome Campus, Hinxton, Cambridgeshire, CB10 1SD UK; Department of Genetics, Stanford University, Palo Alto, CA 94304 USA

**Keywords:** Gene families, Human, Gene symbols, HGNC, BioMart, Genes

## Abstract

The HUGO Gene Nomenclature Committee (HGNC) approves unique gene symbols and names for human loci. As well as naming genomic loci, we manually curate genes into family sets based on shared characteristics such as function, homology or phenotype. Each HGNC gene family has its own dedicated gene family report on our website, www.genenames.org. We have recently redesigned these reports to support the visualisation and browsing of complex relationships between families and to provide extra curated information such as family descriptions, protein domain graphics and gene family aliases. Here, we review how our gene families are curated and explain how to view, search and download the gene family data.

## Background

Grouping human genes together into gene families helps the scientific and clinical community to quickly find related sets of genes in order to plan studies and interpret existing data. There are many resources available that group genes together based on specific product functions such as Carbohydrate-Active enZYmes Database (CAZy) [1], IUPHAR/BPS Guide to Pharmacology [2] and the Nuclear Receptor Resource [3]. There are also homology-based resources such as Ensembl Compara [4] and Panther [5] as well as resources that provide groupings based on domain composition, such as Pfam [6] and InterPro [7]. Additionally, there are ontologies that group human genes together based on function, phenotype or sequence, e.g. Gene Ontology [8], the Phenotype Ontology [9] and the Sequence Ontology [10]. However, not one of these resources utilises the full scope of attributes that can be used to group human genes together. The HUGO Gene Nomenclature Committee (HGNC) [11] is the only manual curation team that specifically groups human genes into families based on a variety of characteristics, whilst also providing further links to other resources for each family member. Therefore, we provide a service that is not available anywhere else.

The core task of the HGNC is to approve unique and informative gene symbols and names for human genes, many of which have been requested directly by researchers via the ‘Gene symbol request form’ [12] on our website. Wherever possible, we name groups of genes together using a common ‘root’ symbol that represents a shared characteristic of that particular set of genes. Root symbols can be based on a variety of attributes including homology, function and structure. We maintain a website, www.genenames.org [13], that provides access not only to HGNC nomenclature but also to related genomic, phenotypic and proteomic information. Our gene symbols are classed as official gene symbols in all major biomedical databases. We release our data nightly, which is freely available via our download tools and services. This allows other resources to update the symbols within their databases according to their release cycles. As other resources have different release cycles, the only way to ensure use of the most up to date gene symbols is via our www.genenames.org resource.

For more than 15 years, the HGNC has provided gene family pages as part of our resource. These pages were originally created to list genes sharing a common root symbol but grouping genes together by root symbol alone provides a restricted list of gene families. We have since expanded this remit and now curate gene families based on many different characteristics with members that do not necessarily share a common nomenclature. How we make these groupings is discussed further below.

Recently, we have restructured our gene family pages to support the visualisation and browsing of complex relationships between families. We now provide visual maps showing these gene family hierarchies and links between pages so that the user may easily navigate between different levels. We have also redesigned our family reports to be of a consistent format and to contain extra curated information such as family descriptions and aliases. The following sections describe these improvements and provide a guide on how to access, view and download HGNC gene family data.

## Curating gene families

Gene families curated by the HGNC can be based on homology, function, the components of a protein complex, shared phenotype or the presence of a particular domain within a set of proteins. All of our gene families are manually curated by HGNC nomenclature advisors. The starting point for a gene family could be a scientific paper as was the case for the ‘SAGA complex’ and the ‘BORCS1 complex’. In other cases, we have worked directly with researchers to curate the gene family, e.g. the ‘Cadherin’ superfamily where the gene family definitions were written by the specialist advisors that helped us to revise the nomenclature and organise the family into a hierarchy. We have over 100 such specialist advisors who help us to decide on the initial membership of our families and the subsequent addition of further members. We also work with specialised external resources to make gene family pages, for example our ‘Blood group antigens’ page is coordinated with the Directory of Red Cell Surface Antigens resource [14] and our ‘Immunoglobulins’ page is coordinated with the IMGT resource [15]. Some families are based on homology using resources such as Ensembl Compara [4], on domain composition following searches of resources such as Pfam [6], or on our own analyses. Large gene family hierarchies are often created based on information from several different sources such as scientific papers, external websites and analysis of domain structure and homology. This was necessary for the development of the ‘G protein-coupled receptors’ superfamily which has a hierarchy with many levels. We also maintain gene family pages that are designed to support a particular community; for example the ‘Long non-coding RNAs’ page allows researchers working on these genes to view gene symbols approved for long non-coding RNA genes that are based on specific research papers.

Once an HGNC curator selects a family to study, we research which genes should be included as family members. As well as protein coding genes, complex families of high variability may include unitary pseudogenes that have functional orthologs in other species or segregating pseudogenes that are protein coding in some individuals and pseudogenes in others. The curator then chooses a suitable name for the family and records the root symbol if one exists. We list commonly used aliases so that our users can find the family even when they do not know the precise name we have chosen. We include key references where inclusion of family members is based on a particular paper or where a paper is particularly relevant to the family members. We try to include a description of the family, which can be sourced from Wikipedia [16], UniProt [17], a publication or can be written by an HGNC curator. If the gene products of the family share a typical domain structure, we select an example gene and the domain structure of this gene product is displayed on the public gene family report. We have the option to include additional comments such as to explain the inclusion of particular family members. We can also include links to relevant external resources such as equivalent gene groups in FlyBase [18] and IUPHAR/BPS guide to pharmacology. If the family is part of a larger hierarchy, we add links to the families that directly precede or succeed the family within the hierarchy.

Each new gene family is automatically assigned a unique numerical gene family ID meaning that our users can store the ID and find the correct gene family even if the family name changes. If we significantly change the current name, we routinely add the old name to the aliases. The family name, ID and aliases as well as the genes within the families can be used to search for gene families using our new search engine which we discuss later in this article.

In June 2012, we had approximately 400 families [19]; and as of October 2015, we have 842 families. The increase in the number of families is due in part to the breakdown of families into subsets as well as the addition of over 200 new families. Now, approximately 45 % of the 39,800 genes with HGNC-approved nomenclature are included in gene families.

## Family report pages

**Fig. 1:**
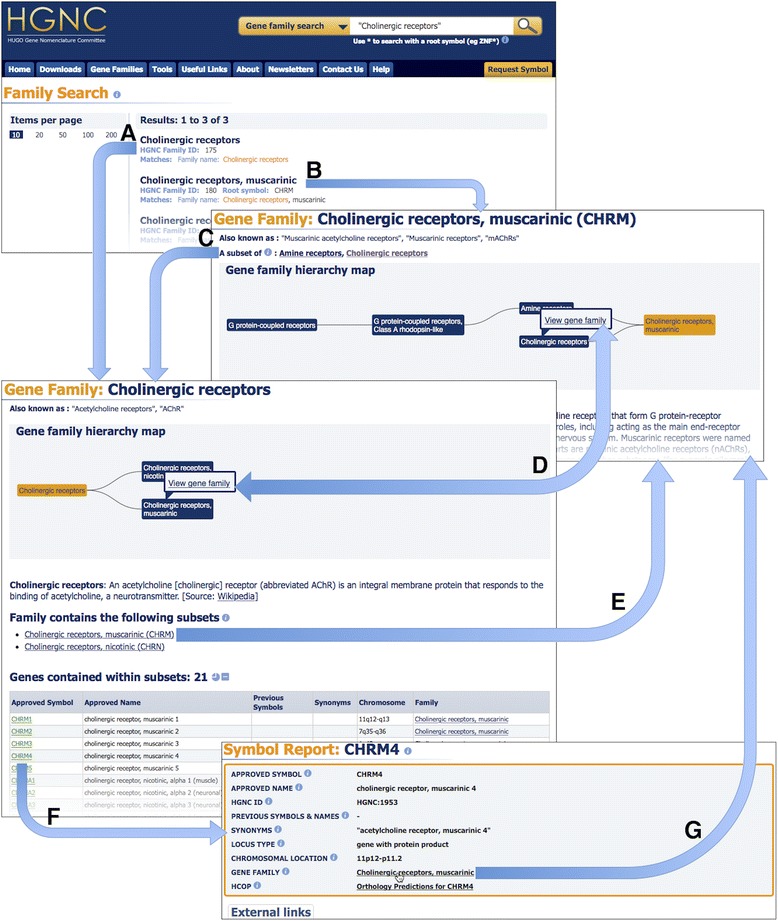
Navigating through the gene family resource. Within the figure, ‘Cholinergic receptors’ was entered in to the search field and three results were returned. Clicking on the first result will navigate to the family ‘Cholinergic receptors’ (*arrow* **a**) whilst the second result navigates to the family ‘Cholinergic receptors, muscarinic’ (*arrow* **b**), a subset of ‘Cholinergic receptors’. It is possible to move through the hierarchy levels by clicking on the ‘A subset of’ link within the subset family (*arrow* **c**), by hovering the cursor over a family of interest within the ‘Gene family hierarchy map’ and clicking ‘View gene family’ within the tooltip (*arrow* **d**), or by clicking on a ‘Family contains the following subsets’ link (*arrow* **e**). Within a gene family report, it is possible to view the gene symbol report for each gene specified in the family by clicking on the approved symbol (*arrow* **f**). The resulting symbol report will have a ‘GENE FAMILY’ field within the top section of the page. It is possible for a gene to be a direct member of several families and if this is the case, they will all be listed within this field. Since the gene *CHRM4* is a direct member of ‘Cholinergic receptors, muscarinic’, this family is listed in the *CHRM4* symbol report and links back to the family (*arrow* **g**)

Each curated gene family has a public gene family report on genenames.org. Gene families may be arranged in complex hierarchies that feature many different levels and each level has its own family report. These levels can be viewed in the ‘Gene family hierarchy maps’ as featured on family report pages (Fig. 1). These show the path through the hierarchy, centred on the current family and only shows those families that are directly related. The full hierarchy can only be seen by selecting the family at the highest level within the map. As well as being a visual representation, the map is also interactive, allowing users to skip to families within the model, highlight paths through the hierarchy and reposition families within the diagram to help in exploring the model.

**Fig. 2:**
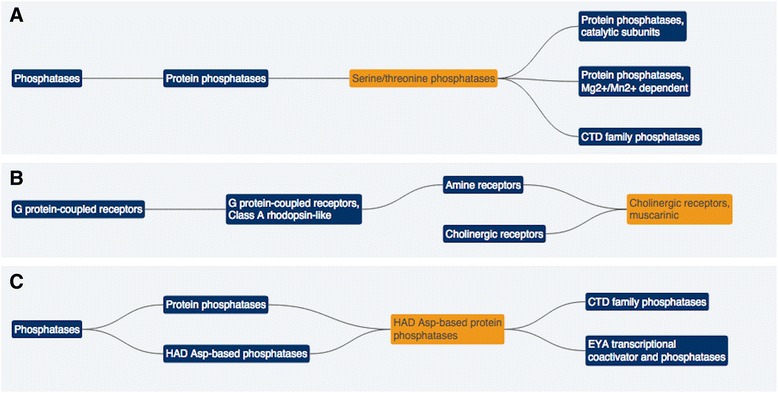
Complex gene family hierarchies. Three hierarchical directed acyclic graphs (DAGs) which show the complex hierarchy levels centred around a highlighted family typical of many gene families within our resource. The gene family hierarchy map for ‘Serine/threonine phosphatases’ (**a**) follows a simple tree-like structure. However, the maps for the ‘Cholinergic receptors, muscarinic’ (**b**) and ‘HAD Asp-based protein phosphatases’ (**c**) families show that our maps are DAGs and do not always follow a tree-like structure

Family hierarchies can form complex structures, e.g. ‘Serine/threonine phosphatases’ which belong to the ‘Protein phosphatases’ family are themselves split into subset families; in this case, the hierarchy model looks like a tree (Fig. 2a). However, a family could be part of more than one hierarchy, for instance the family ‘Cholinergic receptors, muscarinic’ is a subset family of both ‘Amine receptors’ and ‘Cholinergic receptors’ (Fig. 2b). In other cases, a family can be divided into multiple subset families and a particular family could have more than one preceding and succeeding family, e.g. ‘HAD ASP-based protein phosphatases’ family in Fig. 2c. Due to these complexities, it is better to visualise the hierarchies as directed acyclic graphs (DAGs). We redesigned our database and gene family web resource to better reflect the relationships between families and the gene members. This has improved the performance of the resource, created a consistent template for all family report pages and offered improved data downloading choices as discussed below.

**Fig. 3:**
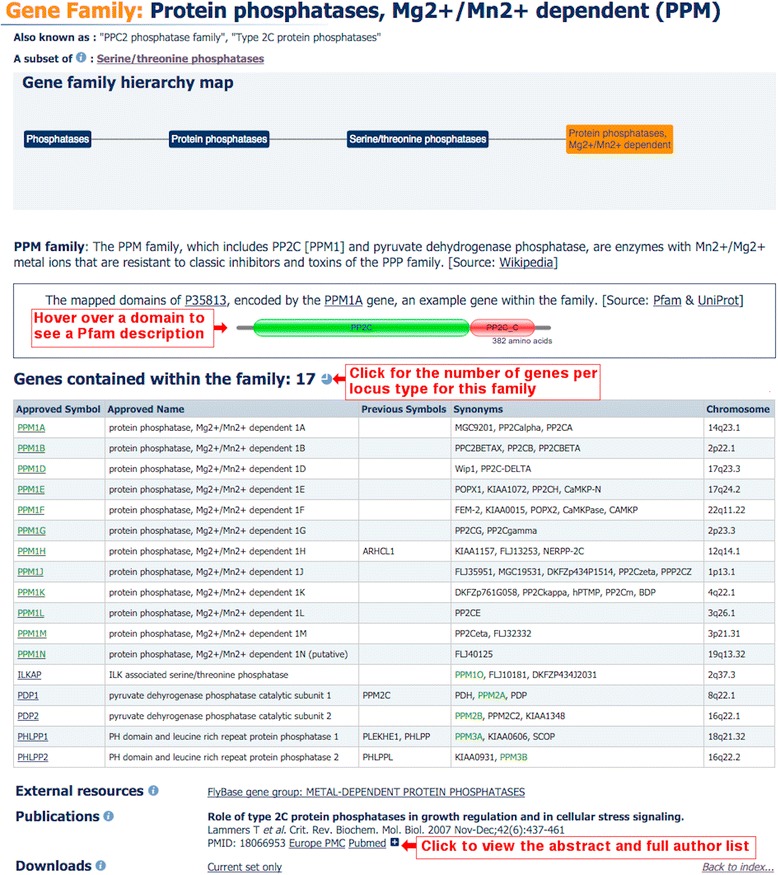
A typical gene family report for a family at the lowest level within a hierarchy

Our gene family reports are arranged in a standard format that allows easy reading and browsing between families. The name of the gene family and, where applicable, the common root symbol are clearly displayed at the top of the page. If present, the gene family aliases are shown beneath the family name as seen in Fig. 3. Where the gene family has a description, the source is clearly displayed in square brackets. As mentioned before, if the members of a gene family share common protein domains, an interactive protein domain graphic for an example gene family member is shown on the page, and the data are sourced from Pfam [6] via UniProt ID (see the graphic for the *PPM1A* gene graphic in Fig. 3).

**Fig. 4:**
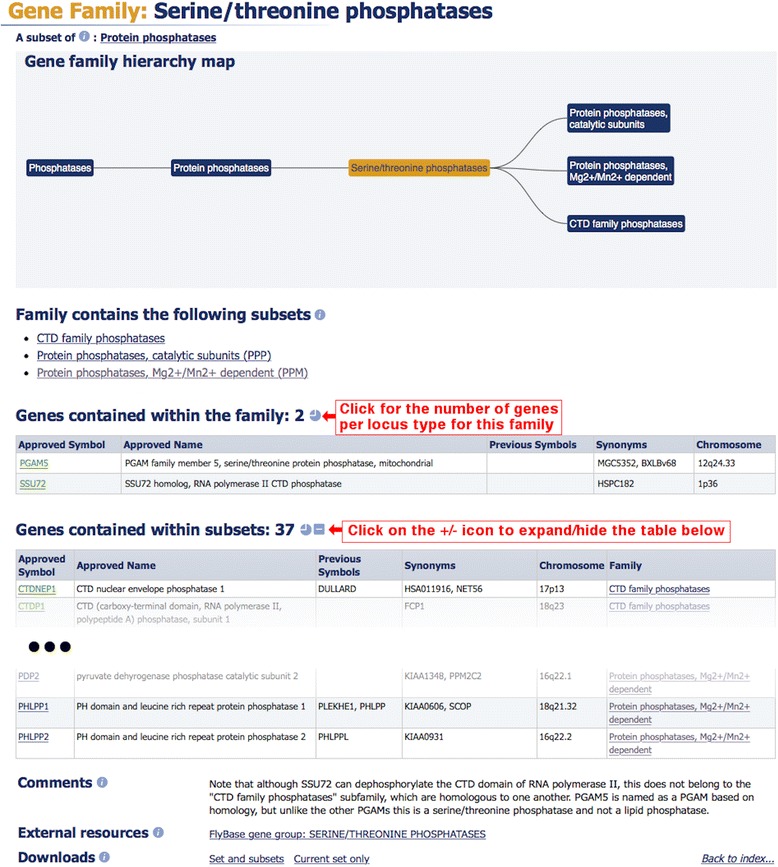
A typical gene family report for a family which contains subset families within a hierarchy

Gene family reports that are not within a hierarchy, or that are at the lowest level of a hierarchy, contain a gene family member table by default. The table of family members is sorted by approved symbol, but where the family shares a root symbol, the members are sorted by that symbol even where it is a synonym or previous symbol, as seen in Fig. 3 in green. Gene family pages that represent higher levels within a hierarchy will display genes by default if those genes do not feature in a specific subset. In the ‘Serine/threonine phosphatases’ example shown in Fig. 4, the default table displays the genes *PGAM5* and *SSU72* and there is a curator comment on the page to give extra information on the presence of these two genes. It is also possible to display all genes that are subsets of ‘Serine/threonine phosphatases’ as shown in Fig. 4. HGNC symbol reports for each gene member within a family can be accessed by clicking on the approved symbol within the gene member table (Fig. 1). These reports will have a field named ‘GENE FAMILY’ which displays the name of the associated family as a hyperlink back to the gene family report, as seen in Fig. 1. A gene can be associated to many families and so the gene symbol report will list the names of all the families of which a gene is a member.

At the bottom of the gene family report, there are a number of fields that only appear if they have been curated, such as external resources and specialist advisors. The publication field lists references relevant to the gene family. By default, a short citation is shown with links to Europe PubMed Central [20] and PubMed [21]; however, it is possible to expand the view to include the abstract and the full author list (Fig. 3).

All gene family members listed within a report can be downloaded from the bottom of each family report. If the family is not within a hierarchy or is at the lowest level of the hierarchy, the ‘Downloads’ section will have the link ‘Current set only’ (Fig. 3), whilst a family containing subset families will have the link ‘Set and subsets’ in the ‘Downloads’ section. Both links will appear if a family contains gene members and subset families (Fig. 4). The ‘Current set only’ link downloads a tab-separated text file that contains the genes that belong to that particular family. The ‘Set and subsets’ link downloads a file that contains all the genes in that family and in associated subset families. Names of subset families are included as a column in the file next to their gene family members.

## Finding gene families

We now have one search application for finding gene families, symbol reports and all other pages on genenames.org using one input box. By default, the search will search across the whole of the site, however, next to the input box, there is a drop down which allows the user to specifically search genes, families or the rest of the site. Information about our search can be found within our ‘Genenames.org: the HGNC resources in 2015’ [11] article.

It is now possible to search for families via the common root/prefix symbol for a family, family name, family alias, family ID, gene name, gene symbol, gene aliases (both symbol and name), previous gene symbols and names, or via the HGNC gene IDs. This should enable our users to find a family of interest even when they do not know the HGNC family name. The results are sorted by a relevance score and each family result will show the family name, the HGNC family ID, the common root symbol (if one exists) and the fields which the query matched, as seen in Fig. 1. Clicking on a result takes you to the report page for that gene family. The search engine can also accept wildcards, quoted phrases and can search a specific field, e.g. ‘family_name: “Olfactory receptors*”’. Our site has a comprehensive ‘Search help’ section [22] that details all the different searchable fields and advanced searching techniques.

We have also added a new dynamic index page where we have taken the list of gene family names and root symbols and placed them into an interactive table which allows our users to filter the columns. The filters work by simply taking the query text entered into the filter box and pattern matching that exact phrase anywhere within the column. At the bottom of the new index page we also show the total number of families and the total number of genes within families. The index page only includes the gene family name and root symbol fields and does not support wildcards. Because of these limitations, we advise our users to search for gene families using the abovementioned search engine.

## Downloading our data

Our gene family data can be downloaded using several different methods. As already mentioned, information about the gene family and the associated genes can be downloaded from within the family report under the ‘Downloads’ section (Figs. 3 and 4).

We also provide two files containing all the genes that are associated to gene families on our ‘Statistics & Downloads’ page [23] with the label ‘HGNC gene family dataset’. One is a text file which has the same format as the download files in the family reports mentioned above. The other file contains the same data but is written as a JavaScript Object Notation (JSON) file. The download files mentioned so far are gene-centric, containing gene families that are found within gene symbol reports. They do not contain information on hierarchical structures but we have a powerful download tool which allows a user to query and select attributes from within our set of gene families and to download all levels of gene family hierarchies. This tool is our new BioMart resource which we have rebuilt to search our gene and gene family data sets using the latest BioMart code [24].

Our new BioMart server [25] has two ‘HGNC mart’ portals, one to download gene-centric data and the other to download gene family centric data. Selecting the family portal will display a form where the user can choose which fields they would like to query, upload a list of IDs and approved symbols, and select which attributes they would like to see within their results table. Submitting the form will create a preview table of results containing the data of interest, and the full data set can then be downloaded as a tab separated text file. The BioMart server can also be queried via REST/SOAP in TSV (tab-separated values), CSV (comma-separated values) or JSON formats as described within the documentation of BioMart 0.9.0 found at BioMart.org [26]. The family mart portal allows the user to download all the gene families and all levels within hierarchies. This is the equivalent of clicking all the ‘Set and subsets’ links within the gene family reports and concatenating all the data into one file. This portal can also link to the genes mart portal, allowing a user to download gene centric attributes found in the gene symbol reports (e.g. gene identifiers from NCBI and Ensembl) for individual gene family members. This means users can associate our gene families to gene information within external gene resources.

The genes mart portal, although gene centric, can also yield gene family data but only the family name and family ID to which the gene belongs. The genes mart cannot travel through the hierarchy of a family and effectively returns similar data to the Custom Downloads tool [27, 28], which has also been updated for the new gene family data. The previous ‘Gene Family Description’ and ‘Gene Family Tag’ fields from the old family data within the tool have been replaced with the ‘Gene family ID’ and ‘Gene family name’ fields. We suggest using our BioMart server in preference to the Custom Downloads tool to download custom built datasets, due to BioMart’s easy to use interface and the choice of gene or family centric data. For further information on how to use our BioMart service please refer to our BioMart Help page [29].

## Conclusions

Our new gene family reports provide more information with new features such as curator comments, alias names, family descriptions, external resource links, publications and protein domain map graphics. Our family reports also show our complex hierarchy models with the new ‘Gene family hierarchy map’ and allow users to navigate easily through the family report pages within the hierarchy. All of our gene and family report pages can be found using our search engine which is quick, simple and powerful, and should be the first port of call for finding gene and gene family reports within our site. Families can also be searched for using our new BioMart server which allows our users to query and tailor a result table for downloading purposes. Our BioMart server is a great one-stop-shop for downloading our data and we also offer a choice to download prepared gene centric files on individual family pages, or entire datasets via our ‘Statistics & Downloads’ page.

By adding new features for searching, visualising and downloading of gene family data, we have made our gene families more accessible and useful to the community. The improvements to the infrastructure of the database have made adding new gene families quicker and easier for HGNC curators. We have more than doubled the number of families within our resource within the last year and we have many more families that we will be adding to our resource in future.

We would very much like to hear from our users for suggestions of new gene families and links to external resources. We would also welcome the input of gene family experts who would like to create a new gene family or collaborate on the content for existing gene families. To contact us, please use our feedback form on our site [30] or email us at hgnc@genenames.org.

## Availability of data and materials

All gene family data are available at http://www.genenames.org. There are no restrictions for using the data.

## Abbreviations

CAZy: Carbohydrate-Active enZYmes Database
CSV: comma-separated values
DAGs: directed acyclic graphs
HGNC: HUGO Gene Nomenclature Committee
JSON: JavaScript object notation
TSV: tab-separated values
